# The effect of hearing protection devices on speech intelligibility of Persian employees

**DOI:** 10.1186/s13104-020-05374-x

**Published:** 2020-11-11

**Authors:** Mina Karami, Mohsen Aliabadi, Rostam Golmohammadi, Morteza Hamidi Nahrani

**Affiliations:** 1 Department of Ergonomics, School of Public Health, Hamadan University of Medical Sciences, Hamadan, Iran; 2 Center of Excellence for Occupational Health, Occupational Health and Safety Research Center, Hamadan University of Medical Sciences, Hamadan, Iran; 3 Center of Excellence for Occupational Health, Research Center for Health Sciences, Hamadan University of Medical Sciences, Hamadan, Iran; 4 Department of Audiology, School of Rehabilitation, Hamadan University of Medical Sciences, Hamadan, Iran

**Keywords:** Noise reduction rating, Speech intelligibility, Hearing protection devices, Work environment

## Abstract

**Objective:**

This study aimed to investigate the effect of hearing protection devices (HPDs) on speech intelligibility in Persian work environments. Three current earmuffs and three earplugs and one of the prototypes of molded earplug were tested on 15 male subjects which were randomly selected. The noise reduction of HPDs was measured based on the Real Ear Attenuation at Threshold (REAT) method. Speech intelligibility during using HPDs was determined based on the speech discrimination score (SDS) at two signal to noise (S/N) ratios (0 and + 5). Data were analyzed using SPSS 22.

**Results:**

The actual to nominal noise reduction rating values were from 47 to 84% for HPDs. At two S/N ratios, no significant differences were observed in speech intelligibility using HPDs (p > 0.05). At S/N ratio = 0, the speech intelligibility descriptively has been only improved by using common earmuffs up to 9.07%. There was a significant difference up to 21.27% in speech intelligibility for proposed molded earplugs at S/N ratio = 0 (p < 0.05). Increasing the HPDs' noise attenuation values led to an increase in speech interference (p < 0.05). The HPDs with the minimum required noise attenuation while maintaining acceptable speech intelligibility should be worn by employees exposed to medium noise levels.

## Introduction

Given that conversation in the work environment is a means of communication, the presence of background noises close to the frequencies of conversation can disrupt communication between employees and even interference with the conversation [1, 2]. Moreover, the use of hearing protection devices (HPDs) by employees for reducing exposure to background noise can also affect their speech intelligibility in the real world. Moreover, the amount of nominal noise reduction rating (NRR) of these devices, which is generally provided by the manufacturing companies in the identification card of these pieces of equipment, is mostly different compared to their actual noise reduction rating [3–5]. Berger et al. proposed the Real-Ear Attenuation at Threshold (REAT) hearing threshold method as the best and most accurate method based on the individuals' subjective responses [6–8].

Previous studies showed that the irregular use of HPDs in work environments can be due to lack of comfort, and interference in conversations. For employees, communication with colleagues and also hearing signals from the equipment and devices is of great importance [9–11]. Nelisse et al. determined that only 64% of employees in that environment used HPDs, and only 20% used them consistently during full shifts [12]. Hashimoto et al. revealed that a decrease in the noise reduction rates of hearing protectors cannot be considered as a factor for improving speech intelligibility [13]. Fernandes et al. showed that at the lowest background noise levels (60 and 70 dBA), HPDs reduced speech intelligibility while the background noise levels were approximately between 80 and 90 dBA and the signal to noise ratio (0, -5, and -10 dB), HPDs improved speech intelligibility [14].

In some jobs with high mental workload such as control room operators and computer-based work in process industries, exposure to medium levels of noise approximately from 60 to 80 dBA can make noise annoyance and discomfort. Therefore, the use of passive HPDs during daily work can be considered to be an accessible solution. However, the consequences of hearing protectors on verbal communication and speech intelligibility of these employees are of great importance. Less attention has been paid about the speech intelligibility result from using HPDs in Persian work environments. This study aimed to investigate the effect of HPDs on speech intelligibility of Persian employees exposed to medium noise emission.

## Main text

### The subject population

**Fig. 1:**
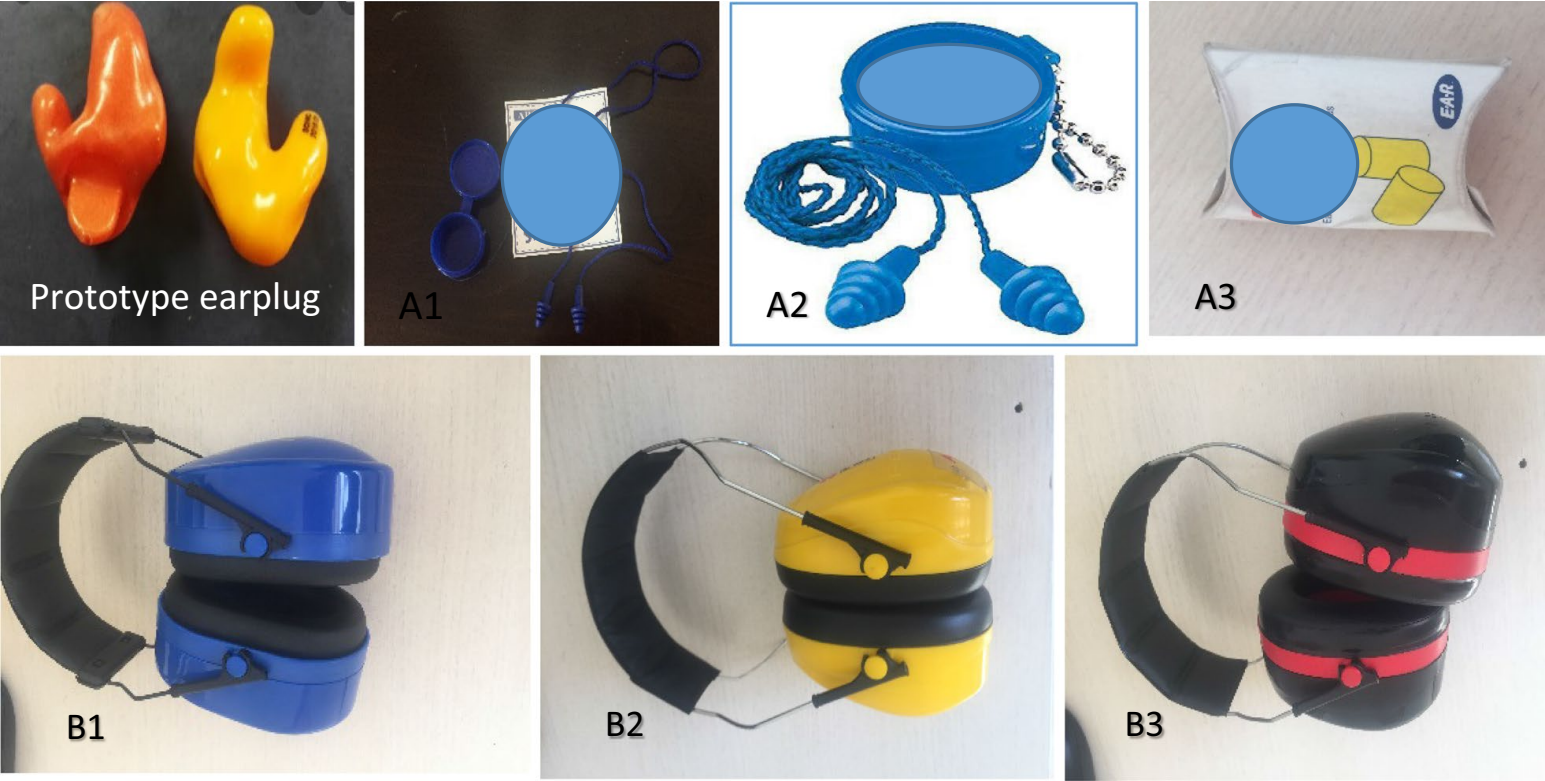
The HPDs types investigated in the current study

In this experimental study, 15 male students of Hamadan University of Medical Sciences with an age range of 18–30 years were randomly selected. Pure tone audiometry was performed for selecting subjects with normal hearing. As shown in Fig. 1, three common commercial earmuffs and three earplug models (one foam formable and two 3-flange pre-molded) with technical specifications from reliable international manufacturers used in the Iranian’s work environments were examined. A prototype of the proposed molded earplug designed based on subjects’ ear shape and size was also tested. The initial ear mold was made using a soft material and the final molded earplug was made from it using silicone materials in the lab. A ceramic filter was placed inside the molded earplug so that it can produce a special channel to allow transmit normal conversation. The inclusion criteria for participating in the study included having normal hearing and vision along with Persian native language.

### Experiment procedure

In each experiment session, based on the REAT method, the hearing threshold of subjects was measured by a reference noise with and without HPDs for 40 min. Next, the speech intelligibility was tested without noise in acoustic room. Finally, the speech discrimination score (SDS) of subjects with and without HPDs was measured at two signal to noise (S/N) ratios for 30 min. As mentioned, based on the REAT method, pure tone audiometery was performed to measure a person's hearing threshold with and without HPDs [15]. In this way, based on the insertion loss of HPDs in the one-octave band frequency spectrum, the actual values of the noise reduction were calculated according to ANSI S3.19-1974 [16–18]. Participants are trained and supervised by the researchers to wear HPDs. The order of testing different types of HPDs was chosen randomly for REAT and SDS tests. All experiments were performed in an acoustic booth as shown in Additional file 1: Fig. S1.

In the next step, using the reference white noise, the ambient background noise was fixed at 70 dBA, using speakers (Pejvak Ava CO) resembles a medium level of noise emission in some offices with high mental workload. Two signal (speech) to background noise ratios (0 and + 5) were considered to relatively resemble the speech level of a speaker at a normal distance to a listener in the mentioned background noise (speech = 70, 75 dBA, noise = 70 dBA).

Speech intelligibility was measured based on the speech discrimination score and using a real two-channel audiometer (Piano model; Inventis CO). According to ISO 8253-3 standard, using a reliable and accurate list of monosyllabic Persian words (25 words), the subjects are asked to repeat the words played by the speaker in the room environment. One of the researchers recorded the answers at a distance of one meter from the listener. Then, the percentage of correctly repeated words is determined [19]. For speech audiometry, the speech of the Persian word from a suitable speaker with normal and clear speech with no particular accent was recorded [20].

### Statistical analysis

Data were analyzed using SPSS software (ver.22, Chicago, IL, USA). The normality of the data was tested using the Kolmogorov-Smirnov test. When data were normally distributed, they were analyzed using the paired sample and student T-tests. The significant relation among some features was analyzed using Pearson correlation. Wilcoxon’s tests was considered for when data distribution is not normal. The significant level for all tests was set at 5%.

## Results

The results showed that the actual to nominal NRR ratio is about 47 to 84%. In the current study, the real to nominal NRR for earplugs was in the range of 47 to 76% (p < 0.05), and for earmuffs, it was in the range of 74 to 84% (p < 0.05). Additional file 1: Fig. S2 represented a comparison between the nominal and actual NRR of all types of studied HPDs. The studied earmuffs showed higher ratios of actual to nominal noise reduction compared with the earplugs. The actual NRR for a proposed prototype earplug was 12.5 dB which was lower than the other studied earplugs. However, this noise attenuation can be more adequate so that there is no overprotection in the medium noise emission environment.

The subjects' speech intelligibility in no background noise, S/N = 0 and S/N =  + 5 conditions were represented in Additional file 2: Table S1. There was a significant difference between the subjects' speech intelligibility without HPDs in these mentioned conditions (p < 0.05). As shown in Additional file 1: Fig. S3**,** Based on the Pearson correlation, a significant correlation is observed between the speech intelligibility in these two signal to noise conditions (r = 0.79 and p < 0.05). Table 1 showed the subjects' speech intelligibility with and without HPDs at S/N = 0. The result showed that there was a significant difference in speech intelligibility for molded filtered earplug (p < 0.05) so that it could increase speech intelligibility by up to 21.27%. Some common earmuffs could only improve speech intelligibility up to 9.07%. Moreover, common earplugs have an intangible effect on speech intelligibility.

**Table 1:** The subjects' speech intelligibility with and without HPDs at S/N = 0

HPDs types	With HPDs (%)	Without HPDs (%)	p value	Difference (%)
Earplug A1	63.20 ± 1.30	62.93 ± 2.90	0.71	0.27
Earplug A2	63.47 ± 6.30	62.93 ± 2.90	0.86	0.54
Earplug A3	63.98 ± 4.80	62.93 ± 2.90	0.88	1.05
Earmuff B1	64.00 ± 3.70	62.93 ± 2.90	0.64	1.07
Earmuff B2	72.00 ± 2.40	62.93 ± 2.90	0.58	9.07
Earmuff B3	72.00 ± 3.40	62.93 ± 2.90	0.13	9.07
Prototype earplug	84.20 ± 3.50	62.93 ± 2.90	*0.04*	21.27

Italic value inside the table indicating significant difference (p < 0.05)

Table 2 showed the subjects' speech intelligibility with and without HPDs at S/N =  + 5. The results showed that there were no significant differences in speech intelligibility in all examined HPDs (p > 0.05). The results showed that HPDs have not notable effect on speech intelligibility at S/N =  + 5.

**Table 2:** The subjects' speech intelligibility with and without HPDs at S/N =  + 5

HPDs types	With HPDs (%)	Without HPDs (%)	p value	Difference (%)
Earplug A1	74.13 ± 4.40	72.00 ± 2.70	0.15	2.13
Earplug A2	72.80 ± 2.10	72.00 ± 2.70	0.71	0.80
Earplug A3	72.10 ± 1.10	72.00 ± 2.70	0.70	0.10
Earmuff B1	73.87 ± 5.60	72.00 ± 2.70	0.10	1.87
Earmuff B2	72.00 ± 5.20	72.00 ± 2.70	0.41	0.00
Earmuff B3	72.00 ± 2.20	72.00 ± 2.70	0.90	0.00
Prototype earplug	75.60 ± 3.60	72.00 ± 2.70	0.10	3.60

As presented in Additional file 1: Fig. S4, the results showed that there was a significant correlation between mean noise reduction values of the earplugs in the frequency range of conversation (250, 500 and 1000 Hz) and the percentage of speech intelligibility at S/N = 5 (r = − 0.37 and p < 0.05). As presented in Additional file 1: Fig. S5, the results showed that there was also a significant correlation between mean noise reduction values of the earplugs and the percentage of speech intelligibility at S/N = 0 (r = − 0.224 and p < 0.05).

## Discussion

Occupational health experts seek to strike a balance between employee hearing protection and their ability to communicate in the work environment with different background noise levels. The actual noise reduction for all studied HPDs was less than their nominal noise reduction, which is consistent with the findings reported by Biabani et al. and Norain et al. [21, 22]. The noise protection data of the tested hearing protectors were relatively similar to the National Institute for Occupational Safety and Health (NIOSH) derating patterns. NIOSH proposed that subtraction of 25% from the manufacturers’ labeled NRR for earmuffs, and 25 to 50% for earplugs [16]. Low quality of the existing HPDs in the real market and the size mismatch to subjects’ anthropometric dimensions are the main reasons for the difference in the actual values to the nominal values. Moreover, the lack of workers’ training about the correct fitting of HPDs can mainly affect the effective noise reduction values in real workplaces.

The participants correctly recognized 98% of the Persian words in silent conditions without HPDs. However, the percentage of identified correct Persian words were reduced to 72% and 62.93% at S/N =  + 5 and S/N = 0, respectively. The result indicated that the higher the background noise or the more unfavorable signal-to-noise ratio, the worse the speech intelligibility. Some HPDs at S/N = 0 had more effect on improving speech intelligibility compared with at S/N =  + 5. Ljung et al. showed that speech intelligibility was reduced linearly with an increase at the signal to noise by using HPDs, which was consistent with the results of the present study [23].

Fernandez et al. showed at positive signal-to-noise levels, HPDs reduced speech intelligibility and when the signal-to-noise levels were negative, HPDs increase speech intelligibility which was relatively similar to the present study [14]. In industrial environments, where the signal-to-noise level is usually negative, HPDs can considerably improve employees' verbal communication in addition to preventing hearing loss. Dastpak et al. showed that using HPDs can improve speech intelligibility by decreasing signal to noise in the background noises from 75 to 95 dB which were somewhat consistent with the present study [24].

A proposed molded earplug could considerably improve the speech intelligibility compared with the studied traditional HPDs while maintaining the minimum required noise reduction. For reducing the gap between the traditional HPDs' noise reduction and speech communication, some new designs on passive HPDs intelligently may improve communication of employees while also maintaining the minimum required noise reduction. The results showed at S/N =  + 5, the percentage of speech intelligibility more decreased by increasing the noise reductions of earplugs compared to the S/N = 0. Therefore, in favorable signal-to-noise ratio, earplugs with higher noise reduction can more reduce the speech intelligibility. For employees with high mental workload exposed to medium noise levels, the HPDs with the minimum required noise reduction while maintaining acceptable speech intelligibility should be worn.

## Conclusion

Speech communications in work environments are always challenging while wearing hearing protection. The HPDs at S/N = 0 showed a higher effect on improving speech intelligibility of the Persian words compared with at S/N =  + 5. It seems that, if the trend of signal to noise ratio was positive, the HPDs can reduce the ability to understand speech. Moreover, increasing the HPDs' noise attenuation levels led to an increase in speech interference. The HPDs with a minimum required noise reduction while maintaining acceptable speech intelligibility should be worn by employees with high mental workload exposed to medium noise emission. Therefore, some types of the proposed molded earplug without noise overprotection and adequate speech intelligibility can be applied at these workrooms.

## Limitations

The interpretation of the current results is limited to the signal to noise ratios simulated medium noise emission in some workrooms such as computer workstations in industrial control rooms. It is proposed that employees' speech intelligibly be measured while using common HPDs at the other signal to noise ratios such as − 5, − 10, etc.

## Supplementary information

**Additional file 1.** Supplementary Tables.

**Additional file 2.** Supplementary Figures.

## Data Availability

The datasets during and/or analyzed during the current study available from the corresponding author on reasonable request.

## Abbreviations

HPDs: Hearing protection devices
REAT: Real Ear attenuation at Threshold
SDS: Speech discrimination score
NRR: Noise reduction rating
S/N: Signal to noise
NIOSH: National Institute for Occupational Safety and Health
