# Supplementary material for: The effect of hearing protection devices on speech intelligibility of Persian employees
Source: BMC Res Notes. 2020 Nov 11;13:529. doi: 10.1186/s13104-020-05374-x (PMC7659119; doi:10.1186/s13104-020-05374-x)
Supplement: Supplementary file 1 — Additional file 1. Supplementary Tables. [file 13104_2020_5374_MOESM1_ESM.docx]

**
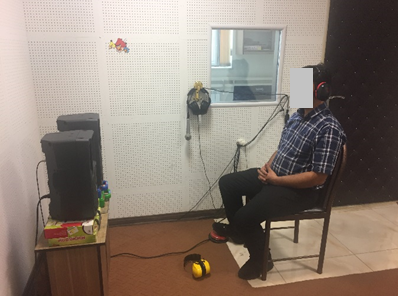
**

**Fig. S1** The experiment set up of the current study.

**Fig. S2** Comparison of the nominal and actual NRR of all types of the studied HPDs.

**Fig. S3**. The scatter plots of speech intelligibility values at S/N=0 compared with the S/N=+5

**Fig. S4**. The speech intelligibility values compared of earplugs' noise reduction (dB) at S/N=5

**Fig. S5** The speech intelligibility values compared to of earplugs' noise reduction (dB) at S/N=0
