# Supplementary material for: The effect of hearing protection devices on speech intelligibility of Persian employees
Source: BMC Res Notes. 2020 Nov 11;13:529. doi: 10.1186/s13104-020-05374-x (PMC7659119; doi:10.1186/s13104-020-05374-x)
Supplement: Supplementary file 2 — Additional file 2. Supplementary Figures. [file 13104_2020_5374_MOESM2_ESM.docx]

**Table S1.** The subjects' speech ineligibility without HPDs in different conditions.

|  | **Silent** | **S/N=0** | **S/N=5** | **P value** |
| --- | --- | --- | --- | --- |
| Speech Intelligibility (%) | 98.00+1.20 | 62.93+2.90 | 72.00+2.70 | 0.001 |
